# Relationship between diet/exercise and pharmacotherapy to enhance the GLP‐1 levels in type 2 diabetes

**DOI:** 10.1002/edm2.68

**Published:** 2019-05-16

**Authors:** Yuki Fujiwara, Shunsuke Eguchi, Hiroki Murayama, Yuri Takahashi, Mitsutoshi Toda, Kota Imai, Kinsuke Tsuda

**Affiliations:** ^1^ Medical Division, Cardio‐Metabolic Medical Franchise Department Novartis Pharma K.K Tokyo Japan; ^2^ Faculty of Human Sciences Tezukayama Gakuin University Osaka Japan

**Keywords:** diet, exercise, glucagon‐like peptide‐1, pharmacotherapy

## Abstract

The rapid rise in the prevalence of type 2 diabetes mellitus (T2DM) poses a huge healthcare burden across the world. Although there are several antihyperglycaemic agents (AHAs) available including addition of new drug classes to the treatment algorithm, more than 50% of patients with T2DM do not achieve glycaemic targets, suggesting an urgent need for treatment strategies focusing on prevention and progression of T2DM and its long‐term complications. Lifestyle changes including implementation of healthy diet and physical activity are cornerstones for the management of T2DM. The positive effects of diet and exercise on incretin hormones such as glucagon‐like peptide‐1 (GLP‐1) have been reported. We hypothesize an IDEP concept (Interaction between Diet/Exercise and Pharmacotherapy) aimed at modifying the diet and lifestyle, along with pharmacotherapy to enhance the GLP‐1 levels, would result in good glycaemic control in patients with T2DM. Consuming protein‐rich food, avoiding saturated fatty acids and making small changes in eating habits such as eating slowly with longer mastication time can have a positive impact on the GLP‐1 secretion and insulin levels. Further the type of physical activity (aerobic/resistance training), intensity of exercise, duration, time and frequency of exercise have shown to improve GLP‐1 levels. Apart from AHAs, a few antihypertensive drugs and lipid‐lowering drugs have also shown to increase endogenous GLP‐1 levels, however, due to quick degradation of GLP‐1 by dipeptidyl peptidase‐4 (DPP‐4) enzyme, treatment with DPP‐4 inhibitors would protect GLP‐1 from degradation and prolong its activity. Thus, IDEP concept can be a promising treatment strategy, which positively influences the GLP‐1 levels and provide additive benefits in terms of improving metabolic parameters in patients with T2DM and slowing the progression of T2DM and its associated complications.

## 1 INTRODUCTION

Over the past three decades, the world has witnessed a rapid rise in the prevalence of type 2 diabetes mellitus (T2DM) and expected to have 629 million of diabetes patients with 20‐79 years by 2045. This pandemic of T2DM leads not only to health problem such as diabetic‐related complications but also to economic burden which is estimated to be USD 776 billion by 2045 across the globe.^1^ An ageing population, changes in lifestyle, dietary patterns, physical inactivity, obesity and stress are the major contributors for the rise in T2DM. Due to the chronic and progressive nature of the disease, undiagnosed or poorly managed T2DM can lead to increased morbidity and mortality.

Despite the availability of several AHAs and addition of new drug classes to the treatment algorithm, more than 50% of patients with T2DM do not achieve glycaemic targets, suggesting an urgent need for treatment strategies focusing on prevention and progression of T2DM and its long‐term complications.^2^, ^3^ The updated treatment guidelines (2018) recommend making lifestyle changes in diet and physical activity as part of T2DM management. Nutrition therapy with individualized meal plan including energy balance, eating patterns and macronutrient distribution and each nutrition is indicated based on patient's age, body weight, physical activity, baseline HbA1c levels and diabetic‐related complications.^4^ For exercise therapy, patients with T2DM are advised to do 150 min/wk of moderate‐to‐vigorous‐intensity physical activity or 75 min/wk of vigorous‐intensity exercise or interval training for a minimum of 3 d/wk to reduce the risk of T2DM‐related complications.^4^

Landmark studies, which are bases of the guideline, revealed the importance of lifestyle modification including diet and exercise for glycaemic control and prevention of diabetes‐related complications. As intervention for diet, the DIRECT study indicated that Mediterranean and low‐carbohydrate diets may be as effective as low‐fat diets for glycaemic control.^5^ There are several intervention studies focusing on exercise. The IDES study demonstrated that promoting physical activity with aerobic and resistance training improved HbA1c compared to exercise counselling alone.^6^ In addition, the Diabetes Prevention Programme study has shown benefits of lifestyle intervention, at least 150 minutes of physical activity per week to achieve a 7% weight loss, in reducing the incidence of T2DM in subjects at risk of diabetes.^7^ The Look AHEAD study demonstrated that intensive lifestyle intervention through both decreased caloric intake and increased physical activity focusing on weight loss did not reduce the rate of cardiovascular events in overweight or obese adults T2DM patients while achieved better glycaemic control compared to control group.^8^ In the randomized Steno‐2 study of 21 years follow‐up, multifactorial intervention including lifestyle changes (diet, exercise and weight loss) and pharmacotherapy (control of blood glucose, blood pressure and lipid profile) has been shown to reduce the risk of not only microvascular but also macrovascular events in T2DM.^9^ The benefits of an intensive multifactorial treatment approach on the risk of cerebrovascular events and macrovascular complications were demonstrated in Japanese patients in the J‐DOIT3 trial.^10^

Among the available oral AHAs, use of incretin‐based therapies such as DPP‐4 inhibitors has increased recently because of the good glycaemic control with a low risk of hypoglycaemia and weight neutrality associated with these drugs. In Japan, more than 70% of patients with T2DM are being treated with DPP‐4 inhibitors and ~60% of drug‐naïve patients receive DPP‐4 inhibitors as the first‐line treatment.^11^ In view of recent reports demonstrating positive effects of nutrition and diet on incretin hormones such as GLP‐1,^12^ we hypothesize an IDEP (Interaction between Diet/Exercise and Pharmacotherapy) concept by comprehensive literature review. The IDEP concept is the active incretin hormone levels, especially GLP‐1, can be enriched through modifications in the diet, physical exercise and pharmacotherapy, which is further protected from degradation by use of DPP‐4 inhibitors, thereby offers good glycaemic control in patients with T2DM. In this review, we discuss the IDEP concept, focusing on whether diet, exercise and pharmacotherapy can affect GLP‐1 secretion.

## 2 INCRETIN HORMONES

GLP‐1 and glucose‐dependent insulinotropic polypeptide (GIP) are two physiologically important incretin hormones. While GLP‐1 is released within minutes from enteroendocrine L cells by nutritional, hormonal, pharmacological and neural signals, GIP is produced in the duodenal K cells in the proximal small intestine.^13^ GIP and GLP‐1 stimulate pancreatic beta cells to induce insulin secretion. In detail, GIP and GLP‐1 exert their effects by binding to their specific receptors, the GIP receptor and the GLP‐1 receptor. Binding to each receptor activates and increases the level of intracellular cyclic adenosine monophosphate thereby activating protein kinase A (PKA) and exchange protein activated by cAMP2 (EPAC2). PKA and EPAC2 are involved in a wide variety of intracellular events including altered ion channel activity, elevated cytosolic calcium levels and enhanced exocytosis of insulin‐containing granules, all of which contribute to stimulation of insulin section in a glucose‐dependent manner.^14^ Incretin effect accounts for approximately 50% to 70% of total insulin secretion in subjects with normal glucose tolerance; however, in patients with T2DM, the incretin effect is diminished and accounts only for 10% to 40% of insulin secretion. Whether or not the decreased incretin effect is due to T2DM itself, or due to decreased β cell function, remains unclear.^15^ Of the two incretins, we focused on GLP‐1 because it potentiates insulin secretion under hyperglycaemic conditions and reduces blood glucose levels in patients with T2DM along with inhibition of gastric emptying, food intake and glucagon secretion.^16^ In addition to improved glycaemic control and preserving islet β cell mass, GLP‐1 has demonstrated beneficial effects on such as cardiac function and atherosclerotic plaque.^17^

Further, the effect of incretins is dependent on the serum level of the soluble DPP‐4 enzyme. In obese patients with T2DM, the plasma DPP‐4 activity is increased resulting in reduced incretin effect and thereby decreasing the efficacy of DPP‐4 inhibitor therapy.^18^, ^19^ In addition, bariatric surgery aimed at weight loss in individuals with T2DM shown to alter GLP‐1 dynamics resulting in improved secretory response to nutrient intake. This improved nutrient sensing could be due to the increased nutrient load into the jejunum, bypassing duodenum, because of the direct connection to the stomach.^20^ The maximum utilization of GLP‐1 could be achieved by the IDEP concept.

## 3 METHOD TO RETRIEVE ARTICLES

We performed a narrative review as we had three topics integrated in one concept. We searched appropriate articles with the following terms: (Diet OR Food) AND type 2 diabetes AND (DPP‐4 inhibitors AND [GLP‐1 or incretins] or metformin); GLP‐1 AND Food/Diet AND DPP‐4 inhibitors; GLP‐1 AND food/Diet AND metformin; GLP‐1 AND exercise AND metformin; GLP‐1 AND exercise AND DPP‐4 inhibitors and got references both from PubMed and OVID, but as we started working on the draft for individual sections, we have pooled in all the studies that we might have retrieved by hand search as well. And we have also deleted some studies while making the draft short to meet the requirement of the journal.

## 4 DIET THERAPY

Diet therapy is important not only as a fundamental treatment for diabetes but as a method to induce GLP‐1 which collaborates with pharmacotherapy for better glycaemic control. In the following section, we summarize nutrition, food, dietary patterns and a couple of ways to induce GLP‐1 secretion.

### 4.1 Nutrition that induces GLP‐1 secretion

#### 4.1.1 Carbohydrates

Glucose is a potent stimulus for the secretion of incretin hormones. Intestinal glucose sensing is mainly facilitated by the sodium‐dependent glucose transporter (SGLT1), which is present abundantly in the absorptive enterocytes and the apical membranes of L and K cells.^21^ Apart from SGLT1, GLUT2 is essential for glucose sensing and thus plays a role in systemic glucose control.^22^ In addition to SGLT1 and GLUT2, secretion of GLP‐1 in response to glucose may be mediated, at least in part, by the sweet taste receptors (T1R2 + T1R3)^23^ even though, unlike glucose, artificial sweeteners do not trigger insulin, GLP‐1 or GIP release in human.^24^

Sugars including galactose, maltose, sucrose, and 3‐O‐methyl‐D‐glucose (3OMG) and maltitol stimulate GLP‐1 release, whereas fructose, fucose, mannose, xylose and lactose do not stimulate GLP‐1 secretion. A little difference in the molecular structures of sugars could affect stimulation of GLP‐1, however, the mechanism of action is unclear.^25^ Further a non‐caloric sweetener and rare sugar, D‐allulose (D‐psicose), induced GLP‐1 release in animal models.^26^ Short‐chain fatty acids (SCFAs) resulting from fermentation of resistant starch, which include acetate, butyrate and propionate, enhance GLP‐1 levels.^27^, ^28^

#### 4.1.2 Protein and amino acids

Peptide transporter‐1 (PEPT1), calcium‐sensing receptor (CaSR) and GPR‐C6A, which are all highly expressed in L cells, are involved in GLP‐1 release.^29^ L‐arginine, which acts as an insulin secretagogue, increases GLP‐1 levels and improve glucose clearance as shown in a mouse study.^30^ Similarly, glutamine stimulates GLP‐1 secretion in murine GLUTag cells, and Na^+^‐coupled amino acid transporters are thought to play an important role in GLP‐1 secretion.^31^ Amino acids such as glutamine, phenylalanine, tryptophan, asparagine, arginine and the di‐peptide glycine‐sarcosine trigger GLP‐1 secretion through CaSR and PEPT1 receptors.^32^, ^33^

#### 4.1.3 Lipids

Lipids including monounsaturated fatty acids, α‐linolenic acid and docosahexaenoic acid (DHA) act as potent stimulators for GLP‐1 release from L cells.^34^, ^35^ Several G protein‐coupled receptors (GPRs) including GPR40, GPR43, GPR119, GPR120 act as prime sensors for fats, and free fatty acid receptors (FFAR 1/3) play an important role in inducing GLP‐1 release. GPR40 and GPR119 act synergistically and mediate the triglyceride (TG)‐induced secretion of incretins, whereas GPR120 plays a minor role in the regulation of incretin secretion.^36^, ^37^

We summarized the effects of nutrition including carbohydrates, amino acids and lipids and the receptors involved in GLP‐1 levels in vitro and in vivo in Table 1. These experimental evidence indicate that GLP‐1 is secreted in response to multiple factors including a variety of nutrients. However, it is not clear what type of nutrition stimulates GLP‐1 secretion effectively.

**Table 1:** Effect of nutrients on GLP‐1 secretion in vivo and in vitro

Study (Cell lines/animal model)	Stimulus	Concentration and duration	Receptors/transporters	Clinical outcomes (GLP‐1 levels)
In vitro				
Gribble et al 2003^109^ (GLUTag cells)	Glucose and methyl‐α‐glucopyranoside	glucose (0.5 mmol/L) and methyl‐α‐glucopyranoside (100 mmol/L) for 2 h	SGLT1	Control ↑ SGLT1 inhibitor →
Jang et al 2007^110^ (Human enteroendocrine NCI‐H716 cells)	Glucose	Indicated concentrations for 10 min	Taste receptors	Control ↑ T1R3 inhibitor →
Mace et al 2012^33^ (Male Sprague‐Dawley rats, Perfused small intestine)	Gln, Phe, Trp, Asn, Arg	10 mmol/L for 30 min	CaSR	Amino acids ↑ Calhex (CasR inhibitor)→
Diakogiannaki et al 2013^32^ (Primary intestinal epithelial cell)	Gly‐Sar (Glycine‐Sarcosine)	10 mmol/L for 2 h	PEPT1	Wild type ↑ PEPT1^−/−^ →
Tolhurst et al 2012^111^ (Primary intestinal epithelial cell)	SCFA	140 mmol/L for 2 h	GPR43	Wild type ↑ FFAR2/3^−/−^ →
In vivo				
Gorboulev et al 2012^21^ (wild type (C57BL)/Sglt1^−/−^ mice)	d‐glucose	5 min after gavage with _D_‐glucose bolus (6 mg/g body wt)	SGLT1	Wild type ↑ *Sglt* **^−^** ^/^ **^−^** →
Roder et al 2014^22^ (wild type (C57BL)/glut2^−/−^ mice)	Glucose	15 min after oral glucose gavage (4 g/kg)	GLUT2	Wild type ↑ *Glut2* **^−^** ^/^ **^−^** mice ↑
Clemmensen et al 2013^30^ (Diet‐induced obese (DIO) mice, Male C57BL/6 mice)	L‐arginine	15 min after oral gavage of L‐arginine (1 g/kg)	—	Wild type ↑ *Gpr40* ^LacZ/LacZ^→
Edfalk et al 2008^36^ (*Gpr40* ^+/+^/*Gpr40* ^LacZ/LacZ^ mice)	Fat	60 min after oral high‐fat diet	GPR40	Wild type ↑ *Glp1r* KO mice →
Tanaka et al 2008^34^ (Rat GPR120)	α‐linolenic acid	Oral α‐linolenic acid 3 μmol/100 μL for 4 weeks	GPR120	Vehicle → α‐linolenic acid ↑
Shida et al 2013^35^ (diabetic KK‐A^(y)^ mice)	Docosahexaenoic acid (DHA)	Oral DHA (100 nmol/200 μL/40 g body weight) for 4 wk	—	Vehicle → DHA ↑

### 4.2 Foods that affect GLP‐1 secretion

A variety of foods can increase GLP‐1 secretion,^38^ including tortillas,^39^ GFO (glutamine, fibre and oligosaccharide),^40^ probiotics such as *Lactobacillus reuteri,* 41 flaked sorghum biscuits,^42^ vinegar,^43^ extra virgin olive oil,^44^ capsaicin,^45^ low glycaemic index beverages,^46^ soya protein and polydextrose,^47^ fish protein hydrolysate (*Micromesistius poutassou*)^48^ and whey protein^49^ (Table 2). It was reported that saturation of fatty acids and length of fatty acids can have an effect on GLP‐1 release. Olive oil induces GLP‐1 response better than butter in both healthy individuals and in patients with T2DM, suggesting an inverse correlation between GLP‐1 release and saturation of fatty acids.^50^ In addition, fatty acid chains with ≥12 carbons increase GLP‐1 levels more than those with <12 carbons.^51^

**Table 2:** Effect of food on GLP‐1 secretion in humans

Study (population)	Type of nutrient	Duration	GLP‐1 levels	Clinical outcomes (Glucose levels)
Almario et al 2017^112^ (Patients with T2DM)	Whey protein	acute/2 wk	↑	↓
Nobile et al 2016^48^ (Slightly overweight men and women)	Fish protein hydrolysate	90 d	↑	—
Kang et al 2016^45^ (Healthy subjects)	Capsaicin	6 wk	↑	—
Stefoska‐Needham et al 2016^42^ (Healthy subjects)	Sorghum biscuits	—	↑	→
Keller et al 2016^46^ (Healthy young men)	Low glycaemic index beverage	1 wk	↑	—
Soong et al 2016^47^ (Lean subjects)	Soya protein and polydextrose	—	↑	↓
Ames et al 2015^39^ (Healthy subjects)	Tortillas with high insoluble fibre	1 wk	↑	→
Joo et al 2015^40^ (Healthy subjects)	GFO (glutamine, fibre, and oligosaccharide)	—	↑	—
Violi et al 2015^44^ (Healthy subjects)	Extra virgin olive oil	1 mo	↑	↓
Simon et al 2015^41^ (Glucose‐tolerant humans)	*Lactobacillus reuteri*	4 wk	Lean: ↑ Obese: →	—
Dalgaard et al 2004^52^ (Patients with T2DM)	A fat‐rich mixed meal with alcohol	—	↓	→
Mofidi et al 2012^54^ (Overweight or obese males)	Bread made from sourdough	—	↓	→

On the other hand, certain foods can have a negative effect on stimulation of GLP‐1 secretion. For example, consumption of a fat‐rich meal with alcohol in subjects with T2DM results in suppression of GLP‐1 early in the postprandial phase.^52^ Further, reductions in the amount of sucrose in the meal were demonstrated to markedly decrease GLP‐1 secretion and were not restored by addition of sweeteners to compensate for the sweet taste in healthy individuals.^53^ Similarly, bread made from sourdough lowered GLP‐1 response when compared with multigrain or sprouted grain bread in subjects at risk for glucose intolerance^54^ (Table 2).

### 4.3 Dietary patterns that induce GLP‐1 secretion

#### 4.3.1 Effect of a sequence of a dietary regimen intake on incretin secretion

The type of meal, sequence of meal intake and adjustment in calorie consumption can all play a role in maintaining postprandial glucose homoeostasis. It has been reported that consuming fish/meat before rice enhances GLP‐1 secretion compared with consuming rice before fish/meat in both healthy subjects and in patients with T2DM which leads to amelioration of postprandial glucose excursion.^55^ Similarly, a whey preload or protein drink before a meal resulted in increased insulin and GLP‐1 secretion, and decreased gastric emptying, in patients with T2DM.^56^, ^57^ These results suggest that eating carbohydrates later in a meal after protein might be a good strategy to enhance secretion of GLP‐1 and in regulating postprandial glucose.

In patients with T2DM, adjusting the calorie balance in each meal with a high‐energy breakfast and low‐energy dinner has been shown to increase GLP‐1 levels, and decrease prandial hyperglycaemia compared to a high‐energy dinner and lower‐energy breakfast.^58^ Therefore, a change in meal timing with regard to calorie consumption could beneficially modulate GLP‐1 secretion.

#### 4.3.2 Effect of mastication frequency per mouthful and eating speed on incretin secretion

It has been reported that mastication frequency, eating speed and eating duration have an impact on incretin secretion. In healthy individuals, chewing 30‐times per bite increased the endogenous GLP‐1 compared with usual eating, without affecting the concentrations of blood glucose or serum insulin.^59^ Findings from the Watari Study in Japan showed that eating quickly was significantly associated with metabolic syndrome compared with slow eating.^60^ Further, eating the same meal slowly for 30 minutes instead of 5 minutes resulted in higher concentrations of GLP‐1 and peptide YY (PYY) levels in healthy subjects.^61^ Therefore, eating slowly and chewing more could contribute to induce GLP‐1 secretion.

#### 4.3.3 Effect of intermittent fasting vs continuous energy restriction on glycaemic control and incretin secretion

A recent report revealed that intermittent energy restriction is comparable for the reduction of HbA1c with continuous energy restriction in patients with type 2 diabetes.^62^ While a report showed that continuous energy restriction reduced active baseline GLP‐1 from baseline,^63^ there is few report to reveal the effect of energy restriction on GLP‐1 secretion which means further studies are warranted to elucidate effect on GLP‐1 secretion in these important dietary interventions.

## 5 EXERCISE THERAPY

### 5.1 Current situation of exercise therapy for T2DM

Lifestyle modifications, including change in dietary habits and increase in physical activity, are cornerstones for the treatment of T2DM. The effectiveness of exercise, including stretching, aerobic and resistance exercise, depends on the patient's condition such as age, cognitive function and physical activity levels. There is limited evidence on the concrete methods of exercise for optimal glycaemic control and health outcomes, even though adults with T2DM are advised to do these combination exercises. There are some evidence that have shown positive effects of the exercises with several duration time, intensity and methods on glycaemic control. Apart from that, non‐exercise activity thermogenesis (NEAT) can be considered as one of the important energy expenditure activities to get a well control of blood glucose.^64^ Studies have shown that interrupting prolonged sitting with brief 5 minutes bouts of standing or light‐intensity walking can attenuate postprandial increase in glucose levels, insulin, C‐peptide and TG responses in patients with T2DM and improve prandial glucose and insulin responses in patients at high risk for T2DM.^65^, ^66^ Furthermore, in patients with T2DM, interval‐walking training helped maintain insulin secretion and improve insulin sensitivity and disposition index, in contrast to energy expenditure‐matched continuous walking training.^67^ The timing of exercise, for example, after a meal was shown to be effective in reducing glucose and TG levels in obese patients with T2DM who were on standard treatment. The results showed that while resistance exercise before dinner improved postprandial glycaemic control, resistance exercise after dinner improved both postprandial glucose and TG elevation.^68^ In addition, a recent report demonstrated that afternoon exercise is more efficacious than morning exercise at improving blood glucose in T2DM individuals.^69^ Further, calorie restriction and exercise resulted in weight loss, and improved glucose regulation as well as incretin secretion in obese patients with T2DM, suggesting the beneficial effects of combining diet and exercise therapy.^70^

### 5.2 Muscle structure in T2DM

Better understanding of skeletal muscles in terms of type of muscle fibres, size, fibre colour, fatigue resistance, metabolism and insulin sensitivity helps the management of obesity and chronic diseases such as T2DM. For example, slow‐twitch type I fibres with high amount of mitochondria have greater insulin sensitivity compared with the fast‐twitch type II fibres. Skeletal muscles exhibit hypertrophy or atrophy depending on the types of fibres under various conditions, such as ageing and T2DM. Type II fibres have higher susceptibility to atrophy with ageing.^71^ Therefore, muscle fibre twitch transformation could lead to inadequate glycaemic control in elderly patients with T2DM.^72^ On the other hand, if the induction of slow type I skeletal muscle phenotype is possible, it can be a potential treatment for obesity and T2DM.^73^ For example, running exercise inhibit diabetes‐associated type shifting of fibres, a higher percentage of type IIB fibres and a lower percentage of type I and type IIA fibres through an OLTEF rat experiment.^72^

### 5.3 Myokine, muscle‐derived interleukin‐6 (IL‐6) induces GLP‐1 secretion

Skeletal muscle constitutes 30%−40% of body weight and glucose uptake by the skeletal muscles increases by up to 50‐fold during exercise. As exercise‐induced glucose uptake involves a complex molecular signalling that is different from insulin, the glucose uptake is preserved even in insulin‐resistant muscle emphasizing the therapeutic potential of exercise in the management of chronic metabolic disease.^74^ GLUT4 translocation during muscle contraction to sarcolemma and t‐tubules is essential for exercise‐induced glucose uptake. Exercise stimulates glucose uptake through enhancing insulin sensitivity and responsiveness. Muscle contraction, muscle remodelling or exercise training produce several secretory factors such as proteins, growth factors and cytokines (also referred to as myokines) that exert beneficial effects of exercise.^75^ Although the discovery and validation of myokines are underway, a well‐known myokine, IL‐6 plays an important role in accelerating glucose uptake independent of insulin and communicating with central and peripheral organs.^76^ Both type I and type II fibres express the muscle‐derived IL‐6 during exercise, which enhances glucose uptake and fat oxidation through activation of AMP‐kinase and/or phosphatidylinositol 3‐kinase.^77^

Findings from several studies reported the benefits of exercise on GLP‐1 release (Table 3). Both in vitro and in vivo analysis showed that IL‐6 or elevated IL‐6 levels during exercise stimulate GLP‐1 secretion from intestinal L cells and α cells via increased proglucagon and prohormone convertase 1/3 expression.^78^ There are two latest clinical studies demonstrating the role of exercise in GLP‐1 secretion. Firstly, Islam et al,^79^ demonstrated that acute exercise bout in healthy volunteers regulate the appetite via lactate and IL‐6 which leads to GLP‐1 secretion in the aspect of energy homoeostasis. Acute intensive exercise leads to a transient reduction in appetite, suppressing ghrelin which is an appetite‐stimulating hormone and increasing GLP‐1 concentration. Secondly, Eshghi et al^80^ demonstrated the effect of long‐duration (90 minutes), moderate‐intensity exercise bouts in patients with T2DM on IL‐6 and GLP‐1 concentration. The results demonstrated that, IL‐6 levels increased after exercise and GLP‐1 and GIP levels remain increased even after 24 hours of exercise, whereas there was no change in prandial insulin or glucagon levels with no effect on prandial glucose levels. Although few studies have examined the association between exercise and GLP‐1 secretion, more than 30 minutes exercise with high intensity (more than 70% of ventilator threshold) could be required to increase GLP‐1 concentration. However, more studies are required to elucidate the optimal exercise for T2DM.

**Table 3:** Effect of exercise on GLP‐1 secretion

Study (subjects)	Exercise and duration	Myokines involved	GLP‐1 levels	Clinical outcomes
Ellingsgaard et al 2011^78^ (Mice)	Treadmill 90 min	IL‐6 ↑	↑	Glucose ↓
Islam et al 2017^79^ (Healthy subjects)	Running (65% VO_2_max) 30 min after meal (7 kcal/kg, 68% carbohydrate, 15% protein, 17% fat)	IL‐6 ↑	↑	Appetite ↓
Eshghi et al 2017^80^ (Patients with T2DM)	Treadmill (80% ventilatory threshold) 90 min bouts before meal (energy intake: 59% carbohydrate, 19% protein, 22% fat)	IL‐6 ↑	—	Glucose ↓

## 6 PHARMACOTHERAPY

On top of diet and exercise, pharmacotherapy itself is critical to achieve good glycaemic control. In the meantime, there are several medications which induce GLP‐1 not only lowering blood glucose level. In the following section, we summarize antihyperglycaemic agents, antidyslipidaemia agents and antihypertensive agents as GLP‐1 inducers.

### 6.1 Antihyperglycaemic agents

#### 6.1.1 Biguanides

Biguanides are oral medications that reduce plasma glucose via multiple mechanisms.^81^ Advantages of metformin include its high efficacy, low cost, minimal hypoglycaemia risk. Gastrointestinal symptom and lactic acidosis are adverse events to be considered.

Several studies demonstrated the involvement of metformin either directly or indirectly in GLP‐1 secretion (Table 4). It has been hypothesized that metformin can act directly on GLP‐1 biosynthesis by increasing proglucagon expression and processing. Metformin also increases the L cell sensitivity to GLP‐1 secretagogues. Other potential mechanisms by which metformin plays a role in GLP‐1 biosynthesis include as follows.^82^

modulating bile acids (BAs) reabsorption there by stimulating GLP‐1 secretion
modulating gut microbiota, resulting in increased production of either SCFAs or BAs which in turn increases GLP‐1 secretion
reducing lipotoxicity
activating enteric neuropeptide (gastrin‐releasing peptide)
stimulating the parasympathetic nervous system

**Table 4:** Effect of antihyperglycaemic drugs on GLP‐1

Study (population)	Duration	GLP‐1 levels	Mechanism
Metformin			
Kappe et al 2013^113^ (murine GLUTag cell line)	Acute: 0.5 mmol/L for 30 min Long‐term: 0.5 mmol/L for 48 h	Acute: GLP‐1→ Long‐term: GLP‐1↑ Preproglucagon ↓	Metformin direct effect on GLP‐1 secretion
Kim et al 2014^83^ (hyperglycaemic *db/db* mice)	8 wk	GLP‐1↑ in serum GLP‐1 receptor expression in islets ↑	Metformin direct effect on GLP‐1 secretion
Pyra et al 2012^103^ (human enteroendocrine cells NCI‐H716)	Cells were incubated for 2 h at 37°C	GLP‐1↑	Metformin direct effect on GLP‐1 secretion
SGLT1/2 inhibitors			
Powell et al 2013^86^ (high‐fat diet mice)	Single dose	Total and active GLP‐1↑	SGLT1 inhibition
Zambrowicz et al 2013^114^ (Healthy subjects)	12 d	Total and active GLP‐1↑	SGLT1 inhibition
Zambrowicz et al 2013^115^ (Patients with T2DM)	Single dose	Total and active GLP‐1↑	SGLT1 inhibition
Oguma et al 2015^87^ (Zucker diabetic fatty rats)	Single dose	Active GLP‐1↑	SGLT1 inhibition
Ferrannini et al 2014^88^ (Patients with T2DM)	4 weeks	GLP‐1↑	Unknown
α‐GIs			
Hamada et al 2013^116^ (Male Nagoya‐Shibata‐Yasuda (NSY) mice)	4 or 12 wk	Active GLP‐1↑	α‐GI enhancement of bile acid levels
Lee et al 2015^91^ (Kir6.2 knockout mice)	Early phase: 5 min Late phase: 10 and 30 min	Active GLP‐1↑	α‐GIs delay carbohydrate absorption and potentiate GLP1 secretion. Miglitol activates duodenal EC cells, possibly via SGLT3, & potentiates GLP1 secretion via parasympathetic nervous system
Zheng et al 2013^117^ (Patients with T2DM)	24 wk	GLP‐1↑	Large amounts of undigested carbohydrates reach the lower portion of the small intestine which is rich in L cells
Amagai et al 2017^118^ (Suspected late dumping syndrome patients)	Single dose	Total GIP↓ Active GLP‐1↓ in early phase	Inhibiting absorption of carbohydrates in the upper gastrointestinal tract
Pioglitazone			
Zheng et al 2017^92^ (Sprague Dawley rats)	14 wk	Active GLP‐1↑	Improving insulin resistance

In the NCI‐H716 cell line and in mice, metformin has been shown to activate GLP‐1 secretion, probably by altering calcium mobilization and the insulin‐signalling pathway upstream from the canonical Wnt‐signalling. Further, 48‐hour incubation of GLUTag cells with metformin increased GLP‐1 secretion, in addition, the expression of proglucagon was decreased.^83^

The gut microbiota is closely associated with energy metabolism and homoeostasis. Recent report demonstrated that metformin affects gut microbiota in diabetic treatment‐naive T2DM patients which contributes to metformin's antidiabetic effect.^84^ Alteration in microbiota was demonstrated in T2DM patients treated with metformin. The improvement in glucose tolerance was indirectly demonstrated in germ‐free mice transferred human faecal samples obtained from subjects treated with metformin. Metformin increased abundance of *Akkermansia muciniphila*, which could improve glucose homoeostasis, metabolic features. Metformin improved glucose sensing in upper small intestine via changing microbiota and upregulation of SGLT1, which in turn is associated with glucose‐induced GLP‐1 secretion.^85^ Sodium‐glucose cotransporter1/2 inhibitors (SGLT1/SGLT2 inhibitors).

SGLT2 inhibitors reduce plasma glucose by enhancing urinary excretion of glucose then the glucose‐lowering efficacy of these medications is dependent on renal function.^81^ Mycotic genital infections and ketoacidosis are adverse effects to be considered.

LX4211, a dual SGLT1 and SGLT2 inhibitor treatment improved glycaemic control by reducing intestinal glucose absorption, while increasing circulating levels of GLP‐1 and PYY when tested in *Sglt1* ^−/−^ mice and *Sglt2* ^−/−^ mice.^86^ Intestinal SGLT1 inhibition enhances GLP‐1 secretion in normal and diabetic rats. The combined treatment of canagliflozin and teneligliptin increased plasma GLP‐1 levels and improved glucose tolerance compared with either of the monotherapies in Zucker diabetic fatty rats.^87^ Treatment with empagliflozin, a highly potent and selective SGLT2 inhibitor, improved β cell function and insulin sensitivity thereby reducing prandial glycaemia in patients with T2DM in addition to enhanced GLP‐1 response although not significant^88^ (Table 4).

#### 6.1.2 α‐glucosidase inhibitors

α‐glucosidase inhibitors are not listed in the latest ADA/EASD guideline,^81^ while they are drugs that delay the breakdown of carbohydrates in the gut and consequently slow down the absorption of sugars. Gastrointestinal adverse effects are common among α‐glucosidase inhibitors.^89^

In patients with T2DM treated with miglitol or voglibose for 12‐weeks, GLP‐1 responses increased. The probable mechanism underlying increased GLP‐1 levels could be either an increase in GLP‐1 secretion or inhibition of the DPP‐4 enzyme.^90^ Miglitol has been shown to activate duodenal enterochromaffin cells possibly via SGLT3, and potentiates GLP‐1 secretion through the parasympathetic nervous system^91^ (Table 4).

#### 6.1.3 Thiazolidinediones

Thiazolidinediones (TZDs) are oral medications that increase insulin sensitivity and are of high glucose‐lowering efficacy. TZDs are associated with the best evidence among glucose‐lowering medications for glycaemic durability.^81^ Potential adverse effect of TZDs is regarding fluid retention and congestive heart failure, weight gain and bone fracture.

Insulin resistance is associated with impaired GLP‐1 secretion. Use of pioglitazone improved the insulin resistance developed in rats fed with a high‐fat diet as well as increased GLP‐1 secretion^92^ (Table 4). Pioglitazone acts as a potent insulin sensitizer via the nuclear receptor peroxisome proliferator‐activated receptor‐γ (PPARγ). The PPARs are superfamily of ligand‐inducible transcription factors and control the gene expression involved in adipogenesis, lipid metabolism and inflammation. Thiazolidinediones could increase GLP‐1 secretion via PPARγ signalling.^92^

#### 6.1.4 Sulphonylureas

Sulphonylureas lower glucose by stimulating insulin secretion from pancreatic beta cells.^81^ They are inexpensive, widely available and have high glucose‐lowering efficacy. As adverse effects, sulphonylureas are associated with weight gain and risk for hypoglycaemia.

Sulphonylurea seems not affect the secretion of incretin.^93^ Sulphonylurea could uncouple the glucose dependence of the insulinotropic effect of GLP‐1.^94^

### 6.2 Antidyslipidaemia agents

There is evidence that the hypolipidaemic agent, atorvastatin, competitively inhibits the DPP‐4 enzyme which otherwise inactivates the incretin hormones GLP‐1 and GIP. However, this action on DPP‐4 inhibition is not class specific, but is structure specific.^95^ Another antihypercholesterolaemia agent, ezetimibe, stimulated GLP‐1 secretion in high‐fat fed mice not by inhibiting the DPP‐4 enzyme but by the activation of the mitogen‐activated protein (MEK)/extracellular signal‐regulated kinase (ERK) pathway.^96^

### 6.3 Antihypertensive agents

The loop diuretic, bumetanide impaired GLP‐1 secretory response by glycine without affecting glucose‐triggered secretion suggesting that the bumetanide‐sensitive Na‐K‐Cl cotransporter maintains high intracellular Cl^−^concentrations in the GLUTag cells.^97^ The angiotensin receptor blocker (ARB), olmesartan improved glucose intolerance independent of improvements in muscle and/or adipose insulin signalling, which could be attributed to increasing GLP‐1 concentration and pancreatic GLP‐1 receptor expression thereby improving glucose‐dependent insulin secretion.^98^

## 7 IDEP

After reviewing diet, exercise and pharmacotherapy, respectively, we move on to interaction among them. Diet and pharmacotherapy interaction is presented in Table 5. Nutrient preloads including whey protein before a meal, decreased postprandial glycaemic excursions partly due to the slowing of gastric emptying and the stimulating secretion of the incretins GLP‐1 and GIP. In a study with the DPP‐4 inhibitor, vildagliptin, whey preload in metformin‐treated patients enhanced the efficacy of vildagliptin by slowing gastric emptying and reducing postprandial glycaemia, in addition to increase in plasma intact GLP‐1 and GIP.^99^ Similarly, in T2DM patients who consumed a preload drink containing 50 g D‐xylose, level of prandial glycaemia was reduced and the effect of a DPP‐4 inhibitor, sitagliptin, was enhanced.^100^ Further, glycaemic control improved in patients with T2DM taking AHAs and eating glutinous brown rice (GBR) for 8 weeks. Moreover, in patients on DPP‐4 inhibitor therapy, a significant decrease in glycated haemoglobin (HbA1c) levels and glycoalbumin, in addition to increased active levels of the gut hormones GLP‐1 and PYY, were reported, demonstrating additive benefits of GBR.^101^ Other than DPP‐4 inhibitor therapy, metformin has shown positive effects on GLP‐1 levels. It was reported that, mice fed with a HF diet for 12 weeks developed insulin resistance along with increased blood glucose and HbA1c levels, and fasting plasma insulin in conjunction with reduced oral glucose tolerance. However, these effects were reversed with metformin due to improved GLP‐1 responses along with reducing prandial plasma free fatty acids.^102^ The combination of metformin and a prebiotic oligofructose (OFS) improved fat mass, hepatic TG, and decreased plasma DPP‐4 activity and GIP levels in obese rats, thereby improving metabolic outcomes in obesity; however, there was no interactive effect of combining OFS with metformin on GLP‐1 secretion.^103^ Furthermore, recent study showed that a high‐fibre diet improved HbA1c levels as well as increasing GLP‐1 AUC in T2DM patients with acarbose and α‐glucosidase inhibitors. This could be due to acarbose which transforms some of the starch present in the diet into a fibre thereby reducing its digestion and increasing fermentable carbohydrate in the colon.^104^

**Table 5:** Diet‐pharmacotherapy interaction

Study (population)	Type of nutrient	Treatment	Clinical outcomes
Wu et al 2016^99^ (Patients with T2DM)	Whey protein	Metformin + vildagliptin	↓ Glucose excursions ↓ Plasma glucagon ↓ Gastric emptying ↑ Plasma insulin ↑ Plasma GLP‐1
Wu et al 2013^100^ (Patients with T2DM)	D‐xylose preload	Sitagliptin	↓ Glucose excursions ↓ Plasma glucose levels ↓ Insulin‐to‐glucose ratio ↓ Gastric emptying ↑ Plasma GLP‐1
Nakayama et al 2017^101^ (Patients with T2DM)	Glutinous brown rice	Insulin injections with or without oral AHAs	↓ HbA1c levels ↓ Glycoablumin ↓ Postprandial glucose levels ↑ GLP‐1 and PYY
Zhao et al 2018^104^ (Patients with T2DM)	High‐fibre diet (SCFA producers)	Acarbose	↓ HbA1c levels ↑ Plasma GLP‐1
Kappe et al 2014^102^ (Mice study)	High‐fat diet	Metformin	↓ Plasma glucose levels ↓ HbA1c levels ↓ Plasma free fatty acids ↑ Plasma GLP‐1
Pyra et al 2012^103^ (Rat study)	Oligofructose	Metformin	↓ Plasma DPP‐4 activity → GLP‐1 levels ↓ GIP levels ↓ Fat mass

In Japanese patients with T2DM treated with a DPP‐4 inhibitor as monotherapy for 1 year, fat intake, especially saturated fat was shown to be significantly associated with deterioration of HbA1c levels (≥0.4%).^105^

There is little evidence regarding exercise and pharmacotherapy interaction. In contrast to the findings demonstrating exercise improves glycaemic and metabolic parameters in patients with T2DM, one study demonstrated that aerobic exercise did not acutely increase total GLP‐1 and GIP levels in patients with T2DM, however, metformin treatment, independent of exercise, significantly increased total plasma GLP‐1 and GIP concentrations.^106^ On the other hand, a 12‐week aerobic exercise training programme in obese patients with metabolic syndrome reduced plasma DPP‐4 levels, which in turn improved insulin sensitivity and fat oxidation.^107^ Taken together, aerobic exercise could have an effect on reduction of plasma DPP‐4 levels but not GLP‐1 secretion. Resistance exercise which showed increase GLP‐1 secretion through IL‐6 could have an effect on reduction of appetite. Therefore, different types of exercises shown to have different effects on GLP‐1 utility for patients with T2DM. Therefore, the combination of exercise and DPP‐4 inhibitors are compatible for T2DM treatment.

With respect to pharmacotherapy interactions, AHAs such as metformin, SGLT1/2 inhibitors, α‐glucosidase inhibitors and pioglitazone as well as certain antihypertensive agents and lipid‐lowering drugs have a direct or indirect role in increasing GLP‐1 levels. Therefore, the combination of DPP‐4 inhibitors and other AHAs are effective choice for T2DM treatment.

## 8 DISCUSSION/CONCLUSIONS

Diet, exercise and diabetes education alongside pharmacotherapy are crucial for the optimal management of T2DM. Changing the diet regimen and including exercise intervention as part of T2DM management can improve glycaemic control and reduce treatment escalation of AHAs, which otherwise can lead to risk of developing potential adverse effects and puts excessive costs on health care.^108^ The interaction between diet/exercise and pharmacotherapy is limited, and hence we attempted to summarize such studies in the present review.

T2DM is a multifactorial disease affecting multiple pathophysiological pathways, and thus a holistic management of the disease, considering combination of diet and exercise together with pharmacotherapy, can result in better treatment outcomes and improve overall quality of life in patients with T2DM. Despite most antihyperglycaemic drugs enhance GLP‐1 levels, the DPP‐4 enzyme soon degrades the active GLP‐1. However, in the case of treatment with DPP‐4 inhibitors, the effect of enhanced endogenous GLP‐1 levels due to diet or exercise are protected, resulting in prolonged active GLP‐1 levels and good glycaemic control without the need for treatment escalation. Although, there is a lot of evidence regarding effect of diet/exercise and pharmacotherapy on GLP‐1 concentration is available, no direct comparison between studies could be made because of different methodologies used in measuring plasma GLP‐1 levels.

**Figure 1:**
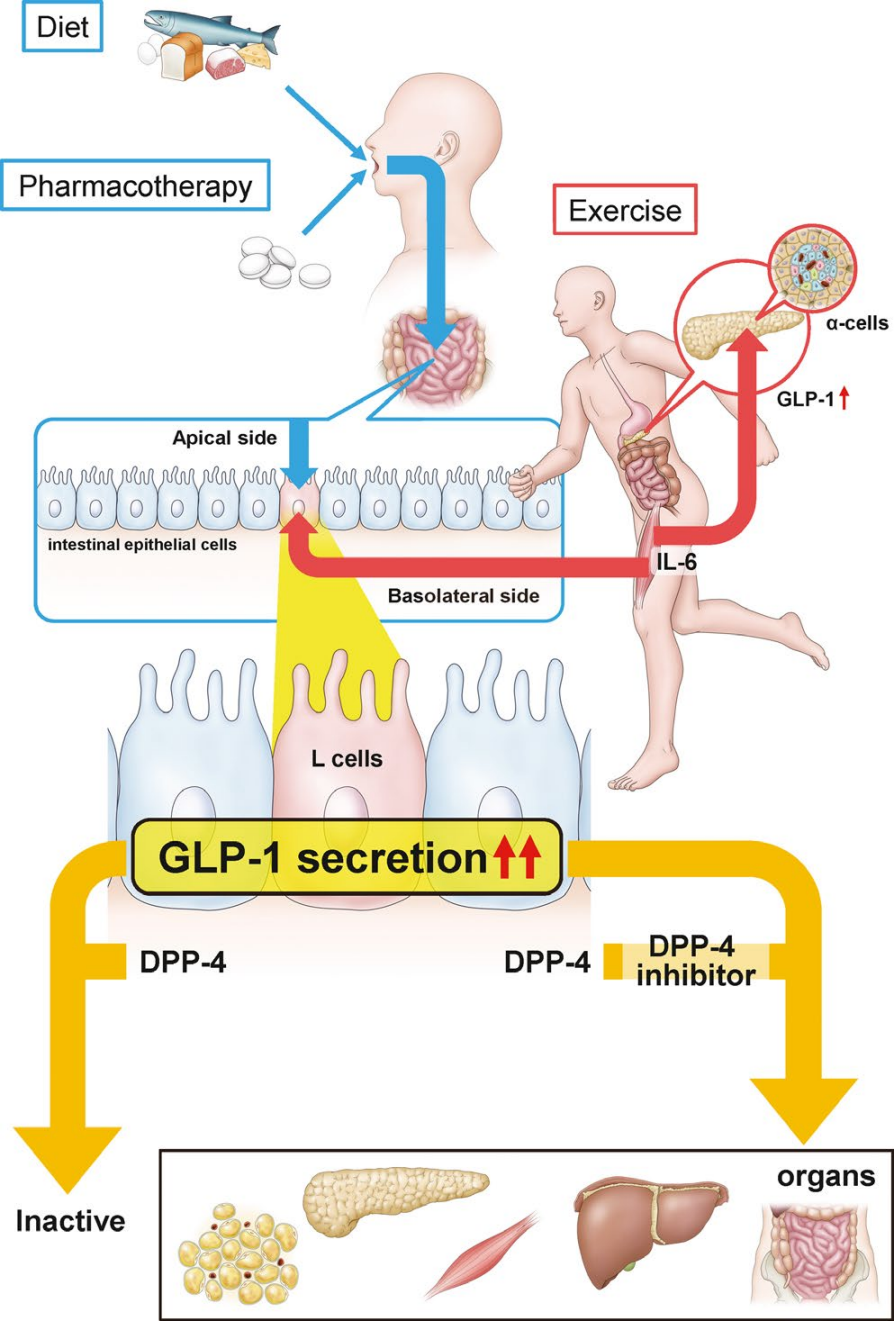
The incretin hormone, glucagon‐like peptide‐1 (GLP‐1) is secreted from intestinal L cells by not only nutrient‐stimulus containing carbohydrate, protein and lipids but also exercise in addition to pharmacotherapy in T2DM patients. The IDEP concept is focused on enhancement of GLP‐1 through changes in the diet, physical exercise and pharmacotherapy. Although GLP‐1 is deactivated by dipeptidyl peptidase‐4 (DPP‐4) except for the neural pathway for the actions of GLP‐1, DPP‐4 inhibitors protect GLP‐1 from degradation. Active GLP‐1 involves positive effects via several mechanisms in pancreas, liver, adipose tissues, muscle and intestines.16 Exercise‐induced interleukin‐6 (IL‐6) concentrations stimulates GLP‐1 secretion from intestinal L cells and pancreatic α cells. Patients with T2DM usually present with comorbidities such as dyslipidemia and hypertension. Therefore, apart from anti‐hyperglycemic drugs, pharmacotherapies with anti‐hyperlipidemia drugs, anti‐hypertensive drugs those have an effect on GLP‐1 release, must be considered for better management of T2DM

To conclude, the IDEP concept can be a promising treatment strategy by positively influencing GLP‐1 levels and providing additive benefits in terms of improving metabolic parameters in patients with T2DM and slowing the progression of T2DM and its associated complications (Figure 1). The IDEP concept further supports and emphasizes the importance of mandating diet therapy and exercise as part of the prescription in order to reduce the disease burden and improve the quality of life in patients with T2DM.

## CONFLICT OF INTEREST

Yuki Fujiwara, Shunsuke Eguchi, Hiroki Murayama, Yuri Takahashi, Mitsutoshi Toda and Kota Imai are employees of Novartis Pharma. Kinsuke Tsuda has served as an advisory board member for Novartis and has received lecture fees from Novartis.

## AUTHOR CONTRIBUTIONS

All the authors researched and analysed the data and were involved in drafting the outline, reviewed all the drafts and approved the final draft of the manuscript.

## ETHICAL APPROVAL

Not applicable.

## Data Availability

All data are included within the manuscript.
